# A prospective study of vaginal trichomoniasis and HIV-1 shedding in women on antiretroviral therapy

**DOI:** 10.1186/1471-2334-11-307

**Published:** 2011-11-03

**Authors:** Linnet N Masese, Susan M Graham, Ruth Gitau, Nobert Peshu, Walter Jaoko, Jeckoniah O Ndinya-Achola, Kishorchandra Mandaliya, Barbra A Richardson, Julie Overbaugh, R Scott McClelland

**Affiliations:** 1 Department of Epidemiology, Box 357236, University of Washington, Seattle, Washington 98195, USA; 2 Department of Medicine, Box 356420, University of Washington, Seattle, Washington 98195, USA; 3 Department of Global Health, Box 358116, University of Washington, Seattle, Washington 98195, USA; 4 Department of Biostatistics, Box 357232, University of Washington, Seattle, Washington 98195, USA; 5 Department of Medical Microbiology, University of Nairobi, P.O. Box 30197-00100, Nairobi, Kenya; 6 Kenya Medical Research Institute, P.O. Box 230-80108, Kilifi, Kenya; 7 Coast Provincial General Hospital, P.O. Box 90231, Mombasa, Kenya; 8 Division of Human Biology, Fred Hutchinson Cancer Research Center, P.O. Box 19024, Seattle, Washington 98109, USA

**Keywords:** *Trichomonas vaginalis*, vaginal infection, antiretroviral therapy, HIV-1, women, Africa

## Abstract

**Background:**

*Trichomonas vaginalis *has been associated with increased vaginal HIV-1 RNA shedding in antiretroviral therapy (ART)-naïve women. The effect of trichomoniasis on vaginal HIV-1 shedding in ART-treated women has not been characterized. We tested the hypothesis that *T. vaginalis *infection would increase vaginal HIV-1 RNA shedding in women on ART, and that successful treatment would reduce vaginal HIV-1 RNA levels.

**Methods:**

We conducted a prospective cohort study including monthly follow-up of 147 women receiving ART in Mombasa, Kenya. Those with *T. vaginalis *infection, defined by the presence of motile trichomonads on vaginal saline wet mount, received treatment with single dose metronidazole (2 g). Test of cure was performed at the next monthly visit. Using the pre-infection visit as the reference category, we compared detection of vaginal HIV-1 RNA before versus during and after infection using generalized estimating equations. A cut-off of 100 HIV-1 RNA copies/swab was used as the lower limit for linear quantitation.

**Results:**

Among 31 women treated for trichomoniasis, the concentration of vaginal HIV-1 RNA was above the limit for quantitation before, during, and after *T. vaginalis *infection in 4 (13% [95% CI 4% - 30%]), 4 (13% [95% CI 4% - 30%]), and 5 (16% [95% confidence interval {CI} 5% - 34%]) women respectively. After adjusting for potential confounding factors, we could detect no difference in the likelihood of detecting vaginal HIV-1 RNA before versus during infection (odds ratio [OR] 1.41, 95% CI 0.23 - 8.79, p = 0.7). In addition, detection of HIV-1 RNA was similar before infection versus after successful treatment (OR 0.68, 95% CI (0.13 - 3.45), p = 0.6).

**Conclusion:**

Detection of vaginal HIV-1 RNA during ART was uncommon at visits before, during and after *T. vaginalis *infection.

## Background

In sub-Saharan Africa, transmission of HIV-1 is predominantly heterosexual [1]. The risk of transmission is likely related to the concentration of virus in genital mucosal secretions, suggesting that reducing genital HIV-1 shedding may reduce infectivity in seropositive individuals [2,3]. Clinical studies provide strong evidence that antiretroviral therapy (ART) leads to rapid and sustained suppression of genital HIV-1 shedding [3,4]. However, this suppression is incomplete [4,5], and persistent genital HIV-1 replication may reflect ongoing risk of transmitting the virus even in individuals on treatment [3].

*Trichomonas vaginalis *infection is highly prevalent in many parts of the world. Among HIV-1 positive individuals, infection with *T. vaginalis *is associated with higher genital HIV-1 levels [6,7]. Successful treatment of trichomoniasis reduces genital HIV-1 levels in antiretroviral-naïve women [7] and men [6,8]. Antiretroviral therapy decreases genital shedding of HIV-1, but there is some evidence that shedding may still be increased in the presence of genital tract infections. For example, a study in HIV-1 seropositive men on ART demonstrated that gonococcal urethritis may activate local genital HIV-1 replication [9].

It is not known whether vaginal trichomoniasis increases HIV-1 shedding in women on ART, or whether treatment of this infection in patients on ART will decrease HIV-1 shedding, potentially decreasing infectivity [10]. To address these questions, we prospectively evaluated the effect of *T. vaginalis *infection on genital HIV-1 shedding in women on ART.

## Methods

### Participants

This study was conducted between March 2004 and December 2008 among HIV-1-seropositive women between 18 and 45 years old in Mombasa, Kenya. The participants were recruited from within a larger cohort of female sex workers in Mombasa [11]. Women eligible for ART initiated a first-line regimen of stavudine or zidovudine, lamivudine, and nevirapine, as recommended by the World Health Organization (WHO) and the Kenyan Ministry of Health Guidelines at the time [12]. All participants gave written informed consent. The study was approved by the ethical review committees of the Kenya Medical Research Institute, the University of Washington, and the Fred Hutchinson Cancer Research Center.

### Clinic procedures

Participants in the ART cohort were asked to return for monthly follow-up visits. During each visit, study nurses completed a standardized interview covering medical, gynecological, and sexual history. The study physician performed a general physical examination and pelvic speculum examination with collection of specimens for laboratory diagnosis of genital tract infections. A Dacron swab was used to collect vaginal secretions for HIV-1 RNA quantitation by placing the swab firmly on the vaginal wall and rolling 3 times between the fingertips. The swab was then placed into a cryovial with freezing medium (70% RPMI, 20% fetal calf serum, and 10% dimethyl sulfoxide with added penicillin, streptomycin, and amphotericin B). Genital samples collected for HIV-1 RNA viral load were stored at -70°C until they were shipped on dry ice to the Fred Hutchinson Cancer Research Center in Seattle for testing. Blood samples were collected every three months for CD4 lymphocyte count.

### Treatment of vaginal infections

Women with symptomatic vaginal conditions (vaginal discharge, itching, or burning) at an examination visit were treated syndromically according to WHO and Kenyan Ministry of Health Guidelines (oral metronidazole 2 g as a single dose combined with clotrimazole 100 mg intravaginal pessaries nightly at bedtime for 7 nights) [13]. All genital specimens were obtained prior to provision of treatment. Patients were asked to return one week after each examination visit for laboratory results. At the results visit, additional treatment was provided if indicated based upon laboratory findings. Those with *T. vaginalis *infection received treatment with single dose oral metronidazole (2 g) only if metronidazole was not dispensed at the examination visit. Samples collected one month post-treatment were used to confirm cure. Women with persistent *T. vaginalis *infection were retreated, and then re-evaluated at the next monthly visit. Participants whose *T. vaginalis *infection resolved after treatment (including retreatment) were included in this study.

### Laboratory methods

HIV-1 serostatus was determined by ELISA (Detect HIV1/2, BioChem Immunosystems, Montreal, Canada). Positive tests were confirmed using a second ELISA (Recombigen, Cambridge Biotech, Worcester, Massachusetts, USA). Quantitation of CD4 lymphocytes was performed using a manual system (Cytosphere, Coulter, Hialeah, Florida, USA) from March 2004 until October 2004, and thereafter by an automated method (FACS Count, Becton Dickinson, Forest Lakes, New Jersey, USA). HIV-1 RNA quantitation was performed using a Gen-Probe viral load assay on vaginal samples (Gen-Probe, San Diego, California, USA) [14].

Vaginal saline wet mounts were examined microscopically at 40X power for the presence of motile *T. vaginalis *parasites and yeast cells or hyphae. Vaginal Gram-stained slides were evaluated for bacterial vaginosis (BV) using Nugent's criteria [15]. Bacterial vaginosis was defined as a Nugent's score of 7-10. Culture for *Neisseria gonorrhoeae *was performed on modified Thayer-Martin media (Difco BD Diagnostics, Oxford, UK). Endocervical samples were also tested for the presence of *N. gonorrhoeae *and *Chlamydia trachomatis *by transcription mediated amplification using the Aptima GC/CT Detection System (Gen-Probe, San Diego, California, USA). The presence of sperm in the genital tract was determined by microscopic examination of Gram stained slides of cervical and vaginal secretions.

### Statistical analysis

Visits before, during and after successful treatment of *T. vaginalis *infection were included in this analysis. Women with concurrent cervical infections were excluded because previous studies demonstrate that cervical infections may influence cervicovaginal HIV-1 shedding [16,17]. We also excluded episodes in which co-infection with both yeast and *T. vaginalis *were present. Cases with concurrent BV were not excluded, as BV commonly coexists with *T. vaginalis*, and our prior prospective studies have not shown a decrease in HIV-1 RNA shedding when women are treated for BV [7]. For this analysis, only treated episodes of *T. vaginalis *infection that were followed by a visit with cure were included. For women with more than 1 consecutive visit with *T. vaginalis *infection, only the first visit with trichomoniasis was included in the analysis.

Our *a priori *sample size calculation was based on the assumption that the majority of women with a genital infection would have quantifiable genital HIV-1 RNA levels. Based on this assumption, 31 cases would allow 90% power to detect a 0.6 log_10 _copies/swab difference in vaginal HIV-1 RNA levels using a two-sided hypothesis test at α = 0.05. We have observed reductions in vaginal HIV-1 RNA greater than 0.6 log_10 _copies/swab with successful treatment of *T. vaginalis *in studies of ART-naïve women [7].

The lower limit for linear quantitation for this assay was 100 copies/ml, which corresponds to 100 copies/swab (swabs were placed in 1 ml of freezing media). Because the majority of vaginal HIV-1 RNA levels were below 100 copies/ml even at the time of *T. vaginalis *infection, we followed our pre-specified alternative analysis plan, which dichotomized the outcome (vaginal HIV-1 RNA) as detectable versus undetectable (< 100 copies/ml). Analyses were performed using SPSS 13.0 (SPSS Inc., Chicago, Illinois, USA) and STATA 9 (StataCorp, College Station, Texas, USA). The primary analysis utilized logistic regression with generalized estimating equations with an exchangeable correlation matrix to compare the presence of detectable HIV-1 RNA in vaginal secretions across the three visits, using the pre-infection visit as the baseline.

Our analysis plan included adjustment for number of months since ART initiation in all models, because increasing duration since ART initiation would be expected to be associated with greater levels of viral suppression, particularly during the first six months of treatment. We then considered adjustment for other potential confounding factors. Adjustment variables considered were adherence to ART (measured by visual analogue scale [VAS], pill count, or pharmacy refill timing), hormonal contraceptive use (use of oral contraceptives, Norplant or depot medroxyprogesterone acetate), presence of concurrent BV, vaginal washing (none, water only, water and soap), and week of the menstrual cycle (week 1-3, week 4 and 5, amenorrheic). We used two approaches to limit the number of potential confounding factors to include in the final model. First, we assessed the change across the three visits for each potential confounding variable, and second, we assessed the association between each variable and the outcome. In the final adjusted model, we only included variables that differed significantly across the three visits or were significantly related to the outcome (p = 0.1).

## Results

Between March 2004 and December 2008, 147 women enrolled in the ART cohort. Of these, 41 acquired *T. vaginalis *infection (incidence, 5.8 per 100 person-years). Ten women were excluded from these analyses. Three did not receive treatment for *T. vaginalis *infection in our clinic because they did not return for results. These women were found to have no *T. vaginalis *present at later examinations. Two women had concurrent cervical infections, and two were lost to follow-up. We also excluded three women who were missing vaginal HIV-1 RNA data (2 at the pre-infection visit and 1 at the infection visit). Thirty-one women were included in the final analysis (Table 1). Their median age was 36 years (interquartile range [IQR] 31 - 38). At ART initiation, the majority were classified as WHO stage 3 or 4 (65%). The median time from ART initiation to the pre-trichomoniasis visit was 10.3 months, IQR (3.6-19.0). Thirty (97%) women remained on first-line ART at the time of their first visit contributing to this analysis, while one woman had changed to a second-line regimen consisting of didanosine, zidovudine, and lopinavir/ritonavir 7 months prior to her infection episode.

**Table 1:** Baseline characteristics of the 31 participants at the pre-infection visit

Characteristic	Median (IQR) or Number (percent)
Demographics	
Age (years)	36 (31 - 38)
Education (years)	7 (7 - 8)
Pregnant	1 (3.2)
Hormonal contraceptive method	7 (22.6)
OCP	1 (3.2)
DMPA	4 (12.9)
Norplant	2 (6.5)
Risk Behavior	
Number of partners in the last week	0 (0 - 1)
Frequency of intercourse in the last week	0 (0 - 2)
Percent condom use in the last week^a^	100 (75 - 100)
Clinical	
WHO HIV staging at ART initiation	
Stage I	3 (9.7)
Stage II	8 (25.8)
Stage III	17 (54.8)
Stage IV	3 (9.7)
CD4 lymphocyte count (cells/ml)	
At start of antiretroviral therapy	126 (68 - 159)
Pre-infection visit^b^	241 (161 - 292)
On stavudine, lamivudine, and nevirapine^c^	30 (96.7)
Time since ART initiation (months)	10.3 (3.6 - 19.0)
ART adherence in the past month	
On-time refills^d^	27 (87.1)
Pill count adherence ≥ 95%	21 (67.7)

IQR, interquartile range.

^a ^Among 15 women who reported sexual intercourse in the last week.

^b ^CD4 count at the pre-infection visit or nearest visit prior to infection was used.

^c ^One woman was on a second-line regimen consisting of lopinavir/ritonavir, didanosine, and zidovudine.

^d ^Late refills were defined as occurring > 48 hours after a refill was due.

The number of women with detectable vaginal HIV-1 RNA before, during, and after *T. vaginalis *infection was 4 (13% [95% confidence interval {CI} 4% - 30%]), 4 (13% [95% CI 4% - 30%]), and 5 (16% [95% CI 5% - 34%]), respectively. For women with detectable vaginal HIV-1 RNA, the median HIV-1 RNA levels before, during, and after *T. vaginalis *infection were 1438 (range 100 - 7223) copies/swab, 451 (range 105 - 928) copies/swab and 770 (range 105 - 29675) copies/swab respectively. Of the eight women with detectable vaginal HIV-1 RNA, one woman had detectable HIV-1 RNA at all three visits, three had detectable HIV-1 RNA at two visits and the other four had detectable HIV-1 RNA at only one visit.

Compared to the pre-infection visit, we were unable to detect differences in genital HIV-1 RNA either during infection (p = 0.96) or following successful treatment (p = 0.81; Table 2). The results were similar after adjusting for potential confounding factors, in sensitivity analyses excluding 5 visits where sperm was detected in genital secretions, and in analyses excluding the participant on second-line ART (data not shown).

**Table 2:** Vaginal HIV-1 RNA detection before, during, and after vaginal trichomoniasis in 31 women

Visit	N (%)	aOR^a ^(95% CI)	P-value	aOR^b ^(95% CI)	P-value
Pre-infection visit	4 (13%)	1.0 (ref)		1.0 (ref)	
Infection visit	4 (13%)	0.97 (0.30 - 3.12)	P = 0.96	1.41 (0.23 - 8.79)	P = 0.71
Post-infection visit	5 (16%)	1.19 (0.28 - 5.05)	P = 0.81	0.68 (0.13 - 3.45)	P = 0.64

aOR, adjusted odds ratio; 95% CI, 95% confidence interval.

^a ^Adjusted for duration on ART.

^b ^Adjusted for BV, late refill, hormonal contraception (yes/no), week of menstrual cycle and duration on ART.

## Discussion

Longitudinal sample collection in this study allowed us to compare genital HIV-1 shedding before, during, and after *T. vaginalis *infection. Among women receiving ART, we could detect no differences in HIV-1 RNA before versus during *T. vaginalis *infection. In addition, we were unable to detect differences in HIV-1 RNA after treatment of vaginal trichomoniasis in this population. Two thirds of the women in this study had undetectable genital HIV-1 shedding before, during, and after an episode of trichomoniasis.

In light of increasing global interest in the secondary HIV-1 prevention benefits of ART [18], it is important to understand whether factors suspected of increasing infectivity in ART-naïve individuals also do so in ART-treated individuals, and thus may be likely to contribute to increased risk of transmission by those receiving treatment. Prior studies have demonstrated that sexually transmitted infections (STIs) are associated with higher genital HIV-1 viral loads in women [7,16,17,19]. However, few studies have examined the effect of STIs or their treatment on genital HIV-1 shedding among women taking ART. Kissinger et al. examined the effect of treating *T. vaginalis *infection on vaginal HIV-1 RNA among ART-naïve and ART-treated HIV-1 positive women [20]. They found that treatment of trichomoniasis was associated with a significant reduction in HIV-1 RNA at 3 months post treatment (odds ratio 0.34, 95% CI 0.12-0.92; p = 0.03). In addition, compared to women not taking ART, women taking ART were less likely to have vaginal HIV-1 RNA shedding (odds ratio 0.25, 95% CI 0.10-0.62, p = 0.01) Based on a prior study of genital HIV-1 shedding in the setting of treatment for *T. vaginalis *in ART-naïve women [7], it is possible that the significant reduction in HIV-1 RNA detection after trichomoniasis treatment observed by Kissinger et al. was driven by the participants who were not taking ART (45%). The study by Kissinger and colleagues did not present results for the ART-treated subset of women separately, so it was not possible to directly compare with our findings. Our present results demonstrate that ART can maintain vaginal HIV-1 suppression in the majority of women even when *T. vaginalis *infection is present.

The results presented here parallel those from recently published data from the same cohort assessing the impact of cervical infections [21] and genital ulcer disease (GUD) on genital HIV-1 shedding [22]. In the cervicitis study, HIV-1 RNA was detected in cervical secretions of 30 women before, during, and after cervicitis at one (3.2%), five (16.1%), and three (9.7%) visits, respectively. In the GUD study of 36 women, HIV-1 RNA was detected before, during and after GUD in cervical secretions from four (11%), one (3%) and six (17%) women, respectively, and in vaginal secretions from three (8%), four (11%) and four (11%) women, respectively. Our present findings add to an expanding evidence base showing that ART generally maintains low or undetectable genital HIV-1 levels even in the presence of STIs.

There were limitations to this study. The sample size was initially calculated based on an assumption that more than half of women would have detectable genital HIV-1 RNA, which would have permitted us to compare the average HIV-1 RNA concentrations at different time points. Since most women actually had undetectable vaginal HIV-1 RNA, we instead used a bivariate outcome based on the presence or absence of vaginal HIV-1 RNA above the threshold for linear quantitation. While this limited the power of our analyses, the results remain useful, as they demonstrate that there were not large increases in either detection or quantity of vaginal HIV-1 RNA associated with episodes of *T. vaginalis *infection. A post hoc power calculation shows that with a sample size of 31, we had 80% power to detect a 5-fold increase in genital HIV-1 RNA detection with *T. vaginalis *infection. A second limitation is that the diagnosis of trichomoniasis by wet mount has limited sensitivity as it relies on visualization of motile protozoa. Because this method is relatively inexpensive, it is still highly relevant in resource-constrained settings such as our study site. A third limitation was the choice to measure only vaginal viral loads. In an earlier study of ART-naïve women, we found that vaginal HIV-1 RNA levels decrease after treatment of *T. vaginalis *infection [7]. Our objective in the present study was to determine if the effect was the same in ART-treated women. Although vaginal secretions are probably the most relevant sample type for a study of genital HIV-1 shedding and *T. vaginalis *infection, we acknowledge that we cannot rule out increases in cervical HIV-1 shedding in women with trichomoniasis. An additional limitation is that these data may not be generalizable to women on other ART regimens. This is particularly relevant in the case of protease inhibitors, which have lower penetration of the genital tract compared to non-nucleoside reverse-transcriptase inhibitors (NNRTIs). However, because NNRTIs are extensively prescribed in first-line regimens throughout the world [12], our findings should have broad applicability.

## Conclusion

In conclusion, we found that detection of vaginal HIV-1 RNA during ART was uncommon at visits before, during and after *T. vaginalis *infection. These findings suggest that the powerful effect of ART on genital HIV-1 shedding may lessen the potentiating effects of vaginal infections on HIV-1 transmission risk.

## Competing interests

The authors declare that they have no competing interests.

## Authors' contributions

RSM, BAR, NP, KM, WJ, and JONA. conceived the question and designed the study. RSM obtained funding for the study. LNM, RSM, SMG, BAR, JO and RG participated in collection and interpretation of the data. LNM, BAR and SMG conducted the data analyses. All authors participated in preparation of the manuscript and approved the final draft for submission.

## Pre-publication history

The pre-publication history for this paper can be accessed here:

http://www.biomedcentral.com/1471-2334/11/307/prepub
